# d-Amino Acid Catabolism Is Common Among Soil-Dwelling Bacteria

**DOI:** 10.1264/jsme2.ME15126

**Published:** 2016-05-11

**Authors:** Atanas D Radkov, Katlyn McNeill, Koji Uda, Luke A Moe

**Affiliations:** 1 Department of Plant & Soil Sciences311 Plant Science Building, University of Kentucky, Lexington, KYUSA 40546-0312; 2 Laboratory of Biochemistry, Faculty of Science, Kochi UniversityKochiJapan

**Keywords:** *Arthrobacter*, rhizosphere, d-amino acids, amino acid catabolism, soil-dwelling bacteria

## Abstract

Soil and rhizosphere environments were examined in order to determine the identity and relative abundance of bacteria that catabolize d- and l-amino acids as the sole source of carbon and nitrogen. All substrates were readily catabolized by bacteria from both environments, with most d-amino acids giving similar CFU counts to their l-amino acid counterparts. CFU count ratios between l- and d-amino acids typically ranged between 2 and 1. Isolates were phylogenetically typed in order to determine the identity of d-amino acid catabolizers. *Actinobacteria*, specifically the *Arthrobacter* genus, were abundant along with members of the α- and β-*Proteobacteria* classes.

Each of the 19 chiral l-amino acids (l-AAs) used in protein synthesis has a mirror image counterpart that is inverted about the α-carbon. These non-proteinogenic AAs are collectively known as d-amino acids (d-AAs). Recent studies have demonstrated the abundance of d-AAs in microbe-rich environments such as the animal rumen, fermented foods, and soil (1, 2, 8, 19, 24). Their origin is typically attributed to bacteria, which are known to employ d-AAs in the synthesis of peptidoglycans as well as other extracellular polymers such as poly-γ-glu and teichoic acids (18). However, it has not yet been determined why diverse d-AAs are synthesized by bacteria. Nevertheless, d-AAs have been shown to affect certain physiological processes in bacteria such as biofilm formation, sporulation, and cell wall modification (10, 12, 20), all of which are important for survival under highly variable physicochemical conditions such as those found in soil.

The region of soil directly surrounding plant roots (the rhizosphere) harbors a diverse chemical milieu owing to plant root exudation, and is known as a hotspot for microbial activity. Some of the available nutrients that attract high numbers of bacteria to this environment are l-AAs, which are prominent components of plant root exudates (14). l-AAs serve not only as potential sources of carbon and nitrogen for rhizobacteria, but also induce chemotaxis and affect root colonization in model-rhizosphere bacteria including *Pseudomonas fluorescens* (17) and *P. putida* KT2440 (13). d-AAs are synthesized from their corresponding l-AAs through enzymatic racemization. Racemization occurs for a number of different reasons, including as part of the l-AA catabolic pathway (18). While l-AA catabolism is an appreciated trait of soil-and rhizosphere-dwelling bacteria, limited information is available on the bacterial capacity for d-AA catabolism.

We herein conducted culture-dependent experiments in an attempt to catalog and characterize bacteria with the ability to catabolize either of 21 AA enantiomer pairs, which include the d- and l-enantiomers of the 19 chiral proteinogenic AAs as well as hydroxyproline (hypro) and ornithine (orn). Soil samples were obtained from the University of Kentucky Horticulture Research Farm (Lexington, KY, USA). Soil was taken from the roots of uprooted, mature sweet corn plants (rhizosphere soil) and from a field left fallow (bulk soil), and approximately 2 g of each was used to perform serial dilutions in Basal Minimal Media (BMM) without any source of carbon or nitrogen (22). l-AAs (one of 21 AAs, 1 mM final concentration) or d-AAs (one of 21 AAs, 1 mM final concentration) (Sigma-Aldrich) were added as the sole carbon and nitrogen source to BMM media, and cycloheximide (250 μg mL^−1^) (Sigma-Aldrich) was added to prevent fungal growth. Agarose, 1.5% (GeneMate, BioExpress), was used as a gelling agent instead of agar in order to minimize non-specific growth. From the 10^−5^ dilution of both samples, 100 μL was spread evenly on each of 6 plates per AA (3 plates for the rhizosphere and 3 for bulk soil) as well as dilute nutrient broth (DNB) plates (0.08 g Difco nutrient broth per L of water [6]). BMM plates without carbon and nitrogen were also prepared in order to estimate non-specific bacterial growth. Plates were incubated at 28°C for two weeks before colony enumeration. Some non-specific growth was noted on nutrient-free BMM plates as colonies that exhibited a starvation-induced phenotype (small, flat, and diffuse morphologies) and these colonies were subsequently excluded from CFU counts on all plates.

Table 1 shows CFU counts for l-AAs and d-AAs from both environments. Colonies were obtained on all d-AA plates, and CFU counts in most cases were not significantly different between the two enantiomers. Central metabolic intermediates, such as the l-enantiomers of glu and asp (as well as their amides gln and asn), and ala were the most commonly utilized substrates. Furthermore, d-ala and d-glu are typically the most abundant d-AAs in microbe-rich environments due to their universal inclusion in peptidoglycans (23), and may be routinely catabolized. Additionally, ala, glu, gln, asn, and asp are the most abundant d-AAs in the tissues of different plant species (3, 24), including maize, and, consequently, may be available in the soil by virtue of exudation or decomposition. Moreover, in cases in which the concentrations of d-AAs have been determined in agricultural soil samples, d-ala, d-asp, and d-glu were the most abundant d-AAs (3).

We calculated two parameters in order to assess variations in growth between AA enantiomer pairs for each soil sample. The difference in (l – d) and ratio (l/d) between l-AAs and d-AAs are shown in Table 1 and were used in order to identify AA enantiomer pairs with the greatest and smallest differences in CFU counts. Significant differences (*p*<0.05) between l-AA and d-AA counts are noted by shading in the table. In both environments, l-glu, l-asn, and l-his conferred a significantly higher number of CFUs and the number of isolates was between two- and four-fold higher on these l-AAs than on their d-AA counterparts (Table 1, shaded cells). Additionally, *trans*-l-hypro, l-ile, and l-asp had the same effect for rhizosphere soil only, while significantly more bulk soil isolates were on plates containing l-ala and l-arg. Differences in CFU counts between other AA enantiomers were not significant, indicating that both enantiomers were readily catabolized.

In order to assess the d-AA and l-AA catabolism potentials of a subset of the cultured isolates, we performed a colony restreak experiment. Colonies were selected from an l-AA plate and were subsequently patched onto a d-AA plate (corresponding to the l-AA plate from which the isolate was taken), as well as onto a fresh l-AA plate. Plates were incubated at 28°C for two weeks. Among each of the 21 AAs, 12 isolates that demonstrated growth on both enantiomers were randomly selected and used to inoculate DNB liquid cultures (2 mL) grown at 28°C with shaking at 220 rpm for two weeks. At the end of the incubation period, glycerol stocks were prepared (25% final glycerol concentration) and stored at −80°C. Each of the glycerol stocks was used directly as a substrate to conduct PCR that targeted the 16S ribosomal RNA gene. PCRs contained DreamTaq 12.5 μL (ThermoScientific), primer 27F (0.5 μM final concentration; 5′ AGAGTTTGA TCMTGGCTCAG 3′), primer 1492R (0.5 μM final concentration; 5′ GGYTACCTTGTTACGACTT 3′) (11), DNase/RNase free water 8 μl, and 2 μl of material from the individual glycerol stock. Cycling conditions were 95°C for 4 min, 95°C for 30 s, 55°C for 30 s, and 72°C for 90 s, repeated 35 times, and finally 72°C for 10 min. PCR products were purified using AMPure magnetic beads (Beckman Coulter) and 50–100 ng of amplicon DNA was used to perform Sanger sequencing using a cycle sequencing kit (BigDye Terminator, v3.1, cycle sequencing kit; Applied Biosystems). Sequencing reactions were purified via AgenCourt CleanSeq magnetic beads (Beckman Coulter) and submitted to the Advanced Genetic Technologies Center at the University of Kentucky. Sequencing data were analyzed through the Ez-Taxon 16S rRNA database (9) in order to obtain the phylogenetic classification of each environmental isolate. Overall, 65% of the collection of 252 isolates gave sequencing data of sufficient quality to determine the taxonomy, and each showed greater than 98% similarity to a known species. Of the 12 isolates for each AA, between 5 and 11 isolates per AA provided sequencing data of sufficient quality to obtain taxonomic information.

Sequences were grouped into four phyla (*Actinobacteria*, *Proteobacteria*, *Bacteroidetes*, and *Firmicutes*; Table 2), 7 classes, 12 orders, 27 families, and 46 genera. Most (92%) of the isolates fell within three classes: *Actinobacteria* (50%), α-*Proteobacteria* (21%), and β-*Proteobacteria* (21%). The most notable result from the taxonomic assignment of the cultured isolates was the abundance of *Arthrobacter* species (32% of all isolates). Isolates within this genus were able to use 18 out of the 21 tested AA enantiomer pairs as the sole source of carbon and nitrogen. Although information in general on the *Arthrobacter* genus is limited, they are metabolically versatile (21) and have been identified as common inhabitants of soil as well as extreme environments (deep subsurface, arctic ice, and sites contaminated with radioactivity or hazardous chemicals) (15). Previous studies on 26 soil isolates from the genus *Arthrobacter* showed that 90% or more were able to catabolize d-ala in addition to other l-AAs (7). Additionally, *A. protophormiae* (DSM15035) catabolizes d-phe, d-leu, and d-met (5), and a d-AA oxidase enzyme from this organism was shown to exhibit the highest activity with d-met, d-lys, d-arg, and d-phe. The metabolic versatility previously noted among members of this genus appears to extend to d-AAs.

Among the most frequently isolated genera in the α-*Proteobacteria* class, *Ensifer* and *Rhizobium* are very closely related (family *Rhizobiaceae*) and belong to the diverse group of plant-nodulating nitrogen-fixing bacteria known as rhizobia. Several of the isolated rhizobia species used both enantiomers of pro, orn, trp, and val as the sole source of carbon and nitrogen. Three genera in the α-*Proteobacteria* class used d-pro as the sole source of carbon and nitrogen (the only other genus that achieved this was *Arthrobacter*).

All of the 13 identified genera within the β-*Proteobacteria* class belonged to the order *Burkholderiales*. Although most of the known plant-nodulating bacteria belonged to the α-*Proteobacteria* class, many isolates of the β-*Proteobacteria* class, specifically of the order *Burkholderiales*, have recently been identified as symbionts of legumes (4, 16). The highest number of genera within the β-*Proteobacteria* class was identified on either enantiomer of phe, as well as gln and ala. While there were four genera in the β-*Proteobacteria* class, there was only one other genus (*Arthrobacter*) capable of using d-phe (Table 2).

Although a large proportion of the identified isolates were part of the three major taxonomic classes discussed above, we identified several genera from the γ-*Proteobacteria* class and δ-*Proteobacteria* class, as well as the classes *Sphingobacteria* and *Bacilli* (Table 2). Based on the results presented here, a number of soil-dwelling bacteria appear to have the capacity to catabolize d-AAs as the sole source of carbon and nitrogen. Our study provides the basis for future work aimed at understanding the impact of d-AAs on the bacterial community in soil and rhizosphere environments.

## Figures and Tables

**Table 1:** Number of isolates at 10^−5^ soil dilution on each AA enantiomer tested.

AA	Rhizosphere soil				Bulk soil			
	
	l	d	l – d	l/d	l	d	l – d	l/d
Ala	58 ± 12	62 ± 4	−4	1	42 ± 5	26 ± 2	17	2
Arg	50 ± 11	43 ± 7	7	1	40 ± 7	24 ± 5	16	2
Asn	75 ± 6	32 ± 10	43	2	43 ± 3	28 ± 4	15	2
Asp	80 ± 5	50 ± 10	30	2	64 ± 8	53 ± 7	11	1
Cys	28 ± 10	21 ± 2	7	1	21 ± 2	16 ± 6	5	1
Gln	62 ± 14	53 ± 6	9	1	55 ± 6	47 ± 8	8	1
Glu	80 ± 8	18 ± 9	62	4	51 ± 7	11 ± 1	40	5
His	43 ± 10	11 ± 3	32	4	28 ± 2	13 ± 3	15	2
Hypro	64 ± 9	27 ± 9	36	2	26 ± 5	19 ± 3	7	1
Ile	66 ± 5	35 ± 10	30	2	38 ± 3	22 ± 11	16	2
Leu	57 ± 5	39 ± 8	18	1	31 ± 5	26 ± 3	5	1
Lys	22 ± 4	32 ± 10	−9	1	20 ± 4	17 ± 4	3	1
Met	21 ± 9	26 ± 5	−4	1	39 ± 9	40 ± 6	−1	1
Orn	46 ± 6	27 ± 12	19	2	41 ± 3	32 ± 15	9	1
Phe	45 ± 17	39 ± 1	6	1	24 ± 7	21 ± 9	3	1
Pro	67 ± 3	71 ± 2	−3	1	30 ± 11	19 ± 4	11	2
Ser	38 ± 14	22 ± 6	16	2	36 ± 7	29 ± 5	7	1
Thr	41 ± 11	48 ± 13	−7	1	41 ± 8	27 ± 7	14	2
Trp	31 ± 3	25 ± 5	6	1	27 ± 7	20 ± 3	8	1
Tyr	42 ± 4	28 ± 9	14	2	27 ± 7	34 ± 9	−7	1
Val	24 ± 7	30 ± 6	−6	1	39 ± 11	36 ± 3	3	1

The average number of isolates is shown (l and d) with the standard deviation. The highlighted cells indicate the enantiomer pairs that had significantly different average values (*p*<0.05). The relative abundance of isolates from rhizosphere soil, as well as bulk soil, was calculated by subtracting the number of isolates counted on d-AA plates from those on l-AA plates for each AA pair (shown in column “l – d”). The ratio of isolates counted on l-AA plates and d-AA plates is shown in column “l/d”.

**Table 2:** Phylogenetic analysis of isolates that catabolized individual l- and d-AA enantiomer pairs as the sole source of carbon and nitrogen.

Phylum	Class	Genus	No. of seq for that genus	Amino acids from which isolates were recovered
*Actinobacteria*	*Actinobacteria*	*Arthrobacter*	53 (32% of total)	ala, arg, asn, asp, cys, glu, gln, his, hypro, ile, lys, orn, phe, pro, ser, thr, trp, tyr
*Actinobacteria*	*Actinobacteria*	*Microbacterium*	9 (5% of total)	cys, his, orn, ser, trp, tyr
*Actinobacteria*	*Actinobacteria*	*Terrabacter*	3 (2% of total)	asp, leu, tyr
*Actinobacteria*	*Actinobacteria*	*Cellulosimicrobium*	2 (1% of total)	met, trp
*Actinobacteria*	*Actinobacteria*	*Agromyces*	2 (1% of total)	his
*Actinobacteria*	*Actinobacteria*	*EU019987_s LAM 22*	1 (1% of total)	ser
*Actinobacteria*	*Actinobacteria*	*GQ396982_s AK1DE1_06H*	1 (1% of total)	val
*Actinobacteria*	*Actinobacteria*	*Nocardioides*	1 (1% of total)	orn
*Actinobacteria*	*Actinobacteria*	*Rhodococcus*	2 (1% of total)	thr
*Actinobacteria*	*Actinobacteria*	*Streptosporangium*	1 (1% of total)	cys
*Actinobacteria*	*Actinobacteria*	*Streptomyces*	4 (3% of total)	met
*Actinobacteria*	*Actinobacteria*	*Micromonospora*	2 (1% of total)	met
*Actinobacteria*	*Actinobacteria*	*Promicromonospora*	1 (1% of total)	thr
*Proteobacteria*	α-*Proteobacteria*	*Ensifer*	7 (4% of total)	ala, glu, pro, trp, tyr, val
*Proteobacteria*	α-*Proteobacteria*	*Rhizobium*	6 (4% of total)	gln, lys, orn, pro, val
*Proteobacteria*	α-*Proteobacteria*	*Pseudaminobacter*	4 (3% of total)	orn, ser, trp
*Proteobacteria*	α-*Proteobacteria*	*Sphingopyxis*	3 (2% of total)	his, leu, lys
*Proteobacteria*	α-*Proteobacteria*	*Bosea*	2 (1% of total)	his, val
*Proteobacteria*	α-*Proteobacteria*	*Candidatus Rhizobium*	2 (1% of total)	glu, ile
*Proteobacteria*	α-*Proteobacteria*	*Mesorhizobium*	2 (1% of total)	his, hypro
*Proteobacteria*	α-*Proteobacteria*	*Ochrobactrum*	2 (1% of total)	asn, pro
*Proteobacteria*	α-*Proteobacteria*	*Ancylobacter*	1 (1% of total)	cys
*Proteobacteria*	α-*Proteobacteria*	*Azospirillum*	1 (1% of total)	tyr
*Proteobacteria*	α-*Proteobacteria*	*Devosia*	1 (1% of total)	asp
*Proteobacteria*	α-*Proteobacteria*	*Shinella*	1 (1% of total)	asn
*Proteobacteria*	α-*Proteobacteria*	*Sphingobium*	1 (1% of total)	ile
*Proteobacteria*	α-*Proteobacteria*	*Sphingomonas*	1 (1% of total)	orn
*Proteobacteria*	α-*Proteobacteria*	*Bradyrhizobium*	1 (1% of total)	thr
*Proteobacteria*	β-*Proteobacteria*	*Pseudoduganella*	7 (4% of total)	ala, arg, asn, hypro, thr
*Proteobacteria*	β-*Proteobacteria*	*Mitsuaria*	4 (3% of total)	ala, his, orn, phe, val
*Proteobacteria*	β-*Proteobacteria*	*Cupriavidus*	4 (3% of total)	ala, gln, phe
*Proteobacteria*	β-*Proteobacteria*	*Pelomonas*	4 (3% of total)	gln, met, val
*Proteobacteria*	β-*Proteobacteria*	*AJ964894_s LF4-45*	2 (1% of total)	asp, phe
*Proteobacteria*	β-*Proteobacteria*	*Rivibacter*	2 (1% of total)	asn, ser
*Proteobacteria*	β-*Proteobacteria*	*Variovorax*	3 (2% of total)	phe, trp
*Proteobacteria*	β-*Proteobacteria*	*Achromobacter*	1 (1% of total)	glu
*Proteobacteria*	β-*Proteobacteria*	*Aquincola*	1 (1% of total)	cys
*Proteobacteria*	β-*Proteobacteria*	*DQ354711_s DR550SWSAEE7*	1 (1% of total)	gln
*Proteobacteria*	β-*Proteobacteria*	*Ideonella*	1 (1% of total)	ser
*Proteobacteria*	β-*Proteobacteria*	*Leptothrix*	1 (1% of total)	leu
*Proteobacteria*	β-*Proteobacteria*	*Massilia*	3 (2% of total)	leu
*Proteobacteria*	γ-*Proteobacteria*	*Pseudomonas*	3 (2% of total)	lys, pro
*Proteobacteria*	γ-*Proteobacteria*	*Moraxella*	1 (1% of total)	cys
*Proteobacteria*	δ-*Proteobacteria*	*Polyangium*	2 (1% of total)	ile, leu
*Bacteroidetes*	*Sphingobacteria*	*Chitinophaga*	1 (1% of total)	pro
*Firmicutes*	*Bacilli*	*Bacillus*	7 (4% of total)	ala, asn, gln, pro
